# 

**Published:** 1882-01

**Authors:** 


### CONTENTS:

ART. I.—Diseases of the Teeth in the International Medical Congress.—
A General Treatment of Irregularities. By Walter H. Coffin............385
The Causes of Irregularities of Position of Teeth.
By Thos. B. Gunning, D. D. S ..................392
The Object and Treatment of Certain Irregularities of the Teeth.
By Oakley Coles ..........................395
ART. II.—The Study of Dental Surgery and the Means Thereto.
By John Tomes, F. R. S., etc.....................400
ART. III.—Death During Chloroform Inhalation ..........................409
ART. IV—The Merits of Soft and Cohesive Gold as a Filling Material.
By A. A. Blount, M. D., D. D. S ...............416
ART. V.—Concerning Dental Colleges. By J. Foster Flagg, D. D. S.................420
ART. VI.—Remarks upon Bonwill's Method of Restoring Crowns upon Roots.
By Dr. E. A. Stebbins.........................423
ART. VII.—A Discovery Rivaling Dr. Jenner's..................................426

### EDITORIAL, ETC.

About Appointment Books..........................................428
Washington City Dental Society.....................................429

### MONTHLY SUMMARY.

Artificial Milk.....................................................429
A Two Thousand Dollar Tooth........................................430
New Treatment of Abscesses.........................................431
About Raising Healthy Women........................................432
A Third Set of Teeth ..............................................432
Largest Book Published ............................................432

